# Editorial: Social and Emotional Influences on Human Development: Perspectives From Developmental Neuroscience

**DOI:** 10.3389/fpsyg.2018.02490

**Published:** 2018-12-06

**Authors:** Nicola K. Ferdinand, Markus Paulus, Tobias Schuwerk, Nina Kühn-Popp

**Affiliations:** ^1^Department of Psychology, Saarland University, Saarbrücken, Germany; ^2^Department Psychology, Ludwig-Maximilians-Universität München, Munich, Germany; ^3^Fakultät für Sozialwissenschaften, Technische Hochschule Nürnberg, Nürnberg, Germany

**Keywords:** developmental science, neuroscience, emotion, social processes, developmental theory

The relevance of emotional and social processes in human development is a central focus in developmental psychology (for an overview see Eisenberg, 2006). Developmental psychology has examined which kind of emotional and social processes play a prominent role in particular phases of development and how their relative impact changes over the life-span. For example, attachment theory has highlighted the role of caregiver-child interaction in the development of emotion regulation capacities during early childhood (cf. Cassidy and Shaver, 2016). Adolescence is known as a period of heightened sensitivity toward peer influences (e.g., Steinberg, 2008). Subjective well-being in the elderly is related to the quality of their social contacts (Pinquart and Sörensen, 2000). Overall, an examination of the developmental significance of particular emotional and social processes has led to a more comprehensive understanding of human development.

Additionally, in the last decades psychology has experienced an increased use of methods derived from neuroscience. More recently, developmental researchers have also started to employ neuroscientific and psychophysiological methods in their empirical investigations. This paved avenues for novel ways to address pertinent research questions. Moreover, this provided novel insights that could not have been obtained with classical research methods. Recent years have also seen an increased employment of neuroscientific methods to explore emotional and social processes, giving reasons to establish new subdisciplines such as *Social Neuroscience* or *Affective Neuroscience*. These topics are also new territory for developmental psychology. This Research Topic brings together novel, cutting-edge neuroscientific research on the impact of emotional and social processes on human development. It highlights the benefits of neuroscientific and psychophysiological methods for fostering our understanding of the role social and emotional processes play during different phases of human development.

## Social Influences on Development

A central focus of many articles has been the social nature of human development. Three studies explored how infants process social stimuli (others' actions and voices) by means of electroencephalography (EEG). Bache et al. combined eye-tracking and EEG to explore how 10-month-old infants process the crawling movement of a same-aged child. Analyses showed differences in sensorimotor mu rhythm between continuous and non-continuous movements. Langeloh et al. explored by means of EEG how 12- to 14-month-olds process usual and unusual actions. They found that the reduction of mu power in frontal regions in response to unusual actions was context dependent, indicating that different social contexts affect how the brain process actions. Zinke et al. employed auditory event-related potentials (ERPs) in 3-month-olds and reliably dissociated components related to short- and long-term memory while the infants were processing familiar and unfamiliar voices.

In their opinion article, Pletti et al. evaluated whether measures relying on pupil dilation allow to discern the motivations underlying early prosocial behavior. They conclude that previous findings do not support claims that early helping is based on intrinsic altruism, but rather on a social motivation to interact with others.

Three further studies explore the role of attachment in human development. Kungl et al. examined preschool-aged children by means of ERPs and found that foster care children show higher arousal to the faces of strangers than control children. Leblanc et al. demonstrate longitudinal associations between early attachment security at 15 months and brain morphometry, mainly gray matter volumes, at 10–11 years. Finally, a longitudinal study by Herd et al. demonstrates the mediating role of inhibition in the interplay of stressful life experiences and secure relationship quality in adolescence.

In their integrative review, Reynolds and Roth discuss empirical findings on the development of attentional biases to faces in the light of current theoretical considerations. Bolenz et al. review the use of computational models to study reinforcement learning under influence of social information throughout development.

Addressing social cognition in autism, a neurodevelopmental condition, Sommer et al. report differences between a group of autistic adults and a comparison group only on the neural, but not on the behavioral level in a false belief task.

## Emotional and Motivational Influences on Development

Three articles examined emotion-cognition interactions during development using pupillary responses, thought to be linked to activation of the noradrenergic system, and ERPs. Hoehl et al. examined attentional biases to threatening stimuli in 6-month-old infants. Pupillary dilation indicated that these biases are likely due to increased arousal and thus hint at an evolved preparedness for developing fear to ancestral threats. Chronaki et al. investigated processing biases related to emotional faces in 6 to 11-year-old children using ERPs. They found that in children with elevated symptoms of anxiety and depression, neural responses to angry faces were increased mostly at later, evaluative processing stages supporting cognitive models of threat perception. Hering et al. studied the influence of emotional material on prospective remembering in younger and older adults. While they found no age-differences on the behavioral level, ERPs revealed that emotional material had differential influences on ongoing processing in younger and older adults, hinting at a negativity bias in younger, but a positivity bias in older adults.

Finally, several articles also looked at motivational aspects and their potential influence on cognitive development. In their review, Kray et al. compared how monetary, cognitive, and social incentives are processed and whether they modulate decision-making and cognitive control during adolescence. Similarly, Ferdinand and Czernochowski reviewed motivational influences on the development of performance monitoring and cognitive control across the adult lifespan. Both reviews highlight the contribution of ERPs on identifying motivational influences on cognitive processes and the usefulness of fMRI to examine the brain substrates underlying these processes. Schmitt et al. provide ERP evidence showing that older adults invest more cognitive resources in preparatory control processes when potential gains are at stake.

## Conclusion

In sum, this Research Topic not only assembles cutting edge work in social and emotional developmental psychology. The explicit discussion of benefits and limitations of neuroscientific methods also offers an overview on potential applications and benefits of these methods in research on social and emotional development.

## Author Contributions

All authors listed have made a substantial, direct and intellectual contribution to the work, and approved it for publication.

### Conflict of Interest Statement

The authors declare that the research was conducted in the absence of any commercial or financial relationships that could be construed as a potential conflict of interest.
